# Supplementary material for: Kinetochores grip microtubules with directionally asymmetric strength
Source: J Cell Biol. 2024 Nov 1;224(1):e202405176. doi: 10.1083/jcb.202405176 (PMC11533501; doi:10.1083/jcb.202405176)
Supplement: Table S2 — lists primers, plasmids, and strains. [file JCB_202405176_TableS2.docx]

| **Supplemental Table 2. Primers Plasmids and Strains.** | |  |  |  |
| --- | --- | --- | --- | --- |
|  |  |  |  |  |
| **For DNA Templates** | |  |  |  |
| **Primer** | **Sequence** | **Purpose** |  |  |
| 50_JDL | /5Biosg/GGTGGTTCTGGTGGTTCTGGTGAATTCCCATTCAATGAAATATATATTTCTTACTATTTC | 180 bp *CEN3 or CEN3* | |  |
| 52_JDL | /5Atto565N/GCTATTCATTGAAAAAATAGTACAAATAAG | 180 bp *CEN3 or CEN3* | |  |
| 30_JDL | ACGAAATTTGGAGTTTGAAACTGAACATAACGTAACAAATcggatccccgggttaattaa | 5' primer to tag Ndc80 | |  |
| 31_JDL | CTGTAGATTGCTCGGGTATTATATATCATTTATTTTATTAgaattcgagctcgtttaaac | 3' primer to tag Ndc80 | |  |
| 10_JDL | GATCTGCAACGTTTGCCTATTCCCGGACATGTACGCCAGGcggatccccgggttaattaa | 5' primer to tag Ctf19 | |  |
| 11_JDL | TAAGCAAGCCGTCCAGTTGGCAATGGCAAATGGAACATCAgaattcgagctcgtttaaac | 3' primer to tag Ctf19 | |  |
| 6_JDL | TCAAAATTCATTTGATGGTCTGTTAGTATATCTATCTAACcggatccccgggttaattaa | 5' primer to tag Ndc10 | |  |
| 7_JDL | TATCCCTATACGAAACAGTTTAAACTTCGAAGCTCCCTCAgaattcgagctcgtttaaac | 3' primer to tag Ndc10 | |  |
|  |  |  |  |  |
| **Plasmid** | **Description** | **Source** |  |  |
| pSB963 | *WT CEN3, 8LacO, TRP1* | Akiyoshi 2009 |  |  |
| pSB972 | *Mutant CEN3 (CCG->AGC CEN mutant), 8 LacO, TRP1* | Akiyoshi 2009 |  |  |
|  |  |  |  |  |
| **Strain** |  |  |  |  |
| SBY19926 | MATa ura3-1::pCSE4-CSE4-XbaI(GFP):URA3 leu2,3-112 his3-11 trp1-1 ade2-1 LYS2+ can1-100 bar1 cse4Δ::KanMX | | | |
| SBY21618 | MATa pDsn1-Dsn1-2D-3FLAG:URA3 Ndc10-GFP:kanMX6 |  |  |  |
| SBY21620 | MATa pDsn1-Dsn1-2D-3FLAG:URA3 Ndc80-GFP:kanMX6 |  |  |  |
| SBY21621 | MATa pDsn1-Dsn1-2D-3FLAG:URA3 Ctf19-GFP:kanMX6 |  |  |  |
| SBY20634 | MATx pDsn1-Dsn1-2D-3FLAG:URA3 Dam1-3GFP:HIS |  |  |  |
