# Supplementary material for: Kinetochores grip microtubules with directionally asymmetric strength
Source: J Cell Biol. 2024 Nov 1;224(1):e202405176. doi: 10.1083/jcb.202405176 (PMC11533501; doi:10.1083/jcb.202405176)

SourceDataFS5. Unedited/uncropped Coomassie-stained SDS-PAGE analyses of the recombinantly purified yeast and human Ndc80 complexes.

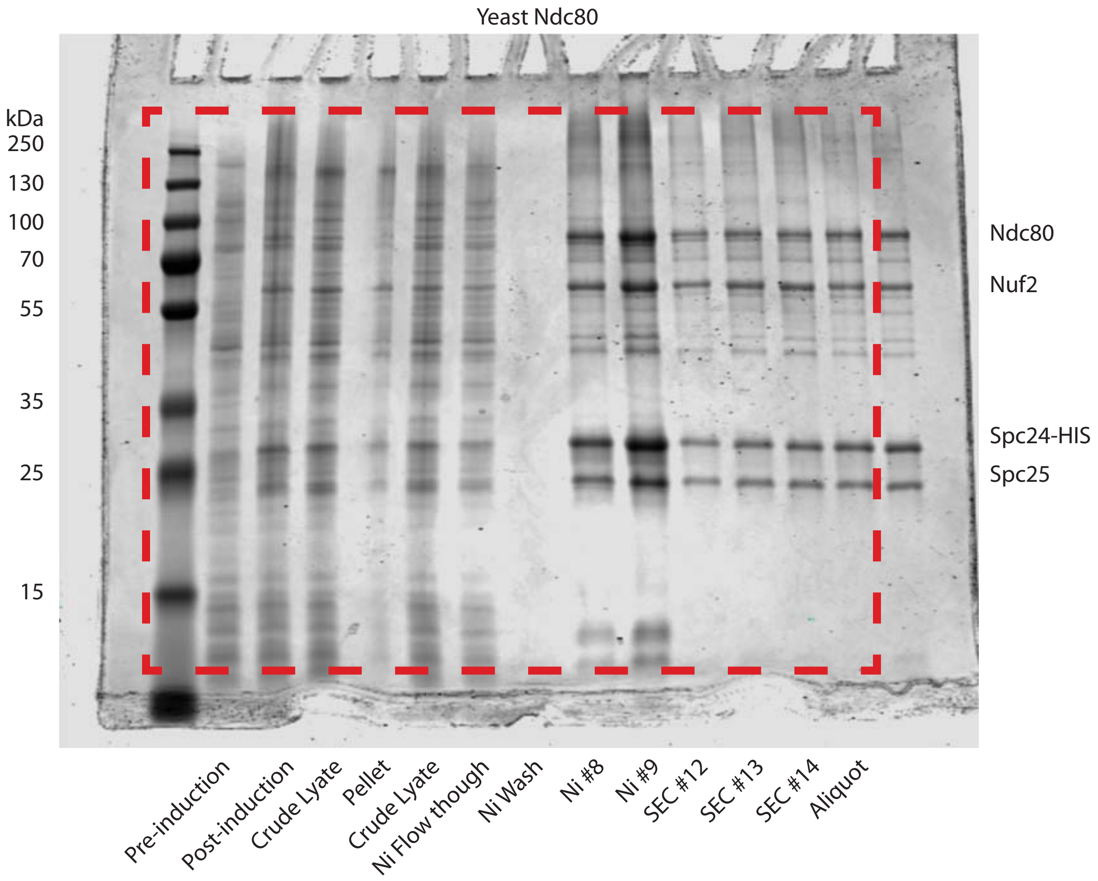

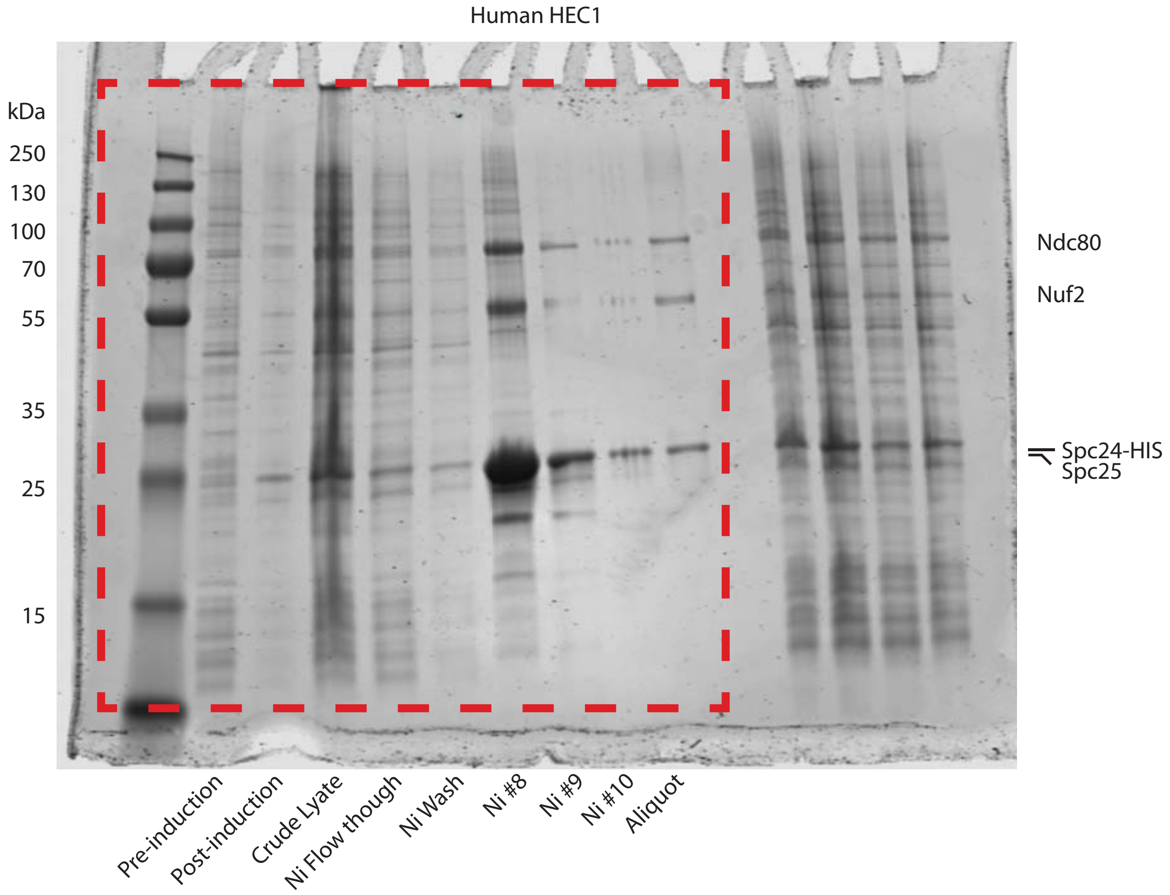

Supplement: SourceData FS5 — is the source file for Fig. S5. [file JCB_202405176_SourceDataFS5.pdf]
